# Uncommon Occurrence of an Air Embolism during the Preanhepatic Phase of an Orthotopic Liver Transplant

**DOI:** 10.1155/2021/6628961

**Published:** 2021-08-09

**Authors:** Klint J. Smart, Saman Yaghoubian

**Affiliations:** Department of Anesthesiology, Westchester Medical Center, 100 Woods Road, Valhalla 10595, NY, USA

## Abstract

Vascular air embolism (VAE) during liver transplantation usually occurs during the dissection phase of the procedure or during liver reperfusion. If this phenomenon occurs, it can cause significant cardiovascular, pulmonary, and neurological complications. Prompt identification of VAE is essential, and the surgeon should be immediately notified. The mainstay treatment is identification and rectification of the source of the air embolus, hemodynamic support, and prevention of further air entrainment. This case report describes the occurrence of a pulmonary air embolism during the preanhepatic phase of an orthotopic liver transplant.

## 1. Introduction

Vascular air embolism (VAE) commonly occurs in liver transplantation while performing vascular anastomosis and during the liver reperfusion phase (inadequate hepatic wash out and anastomotic leaks) [1]. This phenomenon typically develops due to the entrainment of air from the operative field into the venous circulation or from intrahepatic air contained in the donor liver [2]. Air emboli have the potential to enter the right side of the heart and eventually the pulmonary circulation. Air emboli also have the potential to enter the arterial circulation through intracardiac and intrapulmonary shunts which can result in devastating cardiac or neurological complications. We present the case of a VAE during the preanhepatic phase of an orthotopic liver transplant (OLT) due to a large defect in the vena cava.

## 2. Case Description

A 52-year-old patient with a past medical history of decompensated liver cirrhosis secondary to alcohol use underwent an OLT. The patient's symptoms prior to admission included chronic hyponatremia, dyspnea, ascites (requiring multiple paracentesis), bilateral peripheral edema, and altered mental status. The patient had no prior history of cardiovascular or pulmonary comorbidities. Preoperative transthoracic echocardiogram (TTE), electrocardiogram (ECG), and myocardial perfusion imaging were normal. The patient's Model for End Stage Liver Disease-Sodium (MELD-Na) score was 30 and Maddrey's Discriminant Function Score was 67.8 (creatinine, 0.95 mg/dl; bilirubin, 13.1 mg/dl; international normalized ratio, 2.11; sodium, 123 mg/dl; and prothrombin time 24.4 seconds).

Anesthesia was induced with fentanyl (250 mcg), propofol (150 mg), and maintained with sevoflurane. Muscle relaxation was achieved using rocuronium and ventilation was mechanically controlled to maintain an end-tidal carbon dioxide pressure between 30 and 35 mm Hg along with a positive end-expiratory pressure of 5 cm H_2_O. Intraoperative monitors comprised standard American Society of Anesthesiologists monitors and a 20-gauge right radial artery invasive monitor. Pulmonary artery (PA) and central venous pressure (CVP) measurements were obtained continuously via a Swan-Ganz catheter placed through a 9 French multilumen sheath introducer in the right internal jugular vein.

Consistent with our institution's practice, the OLT was done by piggyback technique with the preservation of the recipient's vena cava. After the liver was mobilized in the preanhepatic phase, the short hepatic veins of the liver were identified. A low-normal CVP was maintained to decrease hepatic congestion as well as bleeding from the liver sinusoids and hepatic veins. However, during ligation of these vessels, there was significant bleeding which was controlled by suture ligation. The surgical team anticipated further bleed, and as a result, the native liver hepatectomy was completed on veno-venous bypass at a flow of 3 liters per minute. This required cannulation of the left femoral vein and portal veins providing venous return to the left axillary vein. About three hours into the start of the surgery, during the preanhepatic phase, an inadvertent injury to the inferior vena cava was made. A massive transfusion protocol was initiated as per the hospital's protocol, and the patient was resuscitated and the defect was repaired.

During this time, there was a sudden decline in the patient's end-tidal carbon dioxide from 31 to 11 mmHg. The CVP immediately before this event was 6 mmHg. The pulmonary arterial pressure increased from 25/15 to 43/25 and hypotension ensued. The blood pressure dropped to 67/31, oxygen saturation was 88%, peak airway pressure increased, and sinus tachycardia developed at a rate of 110 beats per minute. An acute pulmonary air embolism was suspected, the operating surgeon was immediately notified, and the field was flooded with normal saline. The fraction of inspired oxygen (FiO_2_) was increased to 100 percent, and the patient required hemodynamic support with 8 mcg/minute of norepinephrine, 2.4 units/hour of vasopressin, and 3 mcg/minute of epinephrine infusions. The patient was placed in reverse Trendelenburg position, and approximately 7 ml of air was aspirated from all 3 ports of the Swan-Ganz catheter.

Over the next 10 minutes, there was gradual improvement in his PA pressure to 18/11, end-tidal carbon dioxide increased to 25 mmHg, oxygen saturation increased to 99%, and the patient became normotensive. ABG revealed a pH of 7.30 along with partial pressures of carbon dioxide and oxygen of 39 mmHg and 253 mmHg, respectively. The patient remained hemodynamically stable thereafter. The patient's total blood loss was 20 liters throughout the case requiring 14 pack red blood cells, 14 units of FFP, 35 units of cryoprecipitate, and 18 units of platelets. The blood product resuscitation was guided by rotational thromboelastometry (ROTEM), estimated blood loss, and hemoglobin levels. At the conclusion of the case, the patient remained intubated and was transferred to the intensive care unit. The patient was extubated on postoperative day (POD) 2, and the postoperative course was complicated by acute renal failure and a small occipital intraparenchymal hemorrhage. On POD16, he was transferred to an acute rehabilitation facility.

## 3. Discussion

There are three main phases that occur during liver transplants: the preanhepatic, anhepatic, and neohepatic phase. Most causes of air embolism thus far that have been reported occur during the surgical dissection phase and during the reperfusion phase. This case report describes the unique development of a pulmonary air embolism during the preanhepatic phase due to a large iatrogenic defect in the vena cava. The preanhepatic phase starts from the initial surgical incision and ends with cross clamping of the portal vein, the inferior vena cava, and the hepatic artery [3]. During the anhepatic and neohepatic phases, air entrainment can occur, while performing surgical anastomosis, during venous bypass, or during liver reperfusion (insufficient hepatic wash out or leaks in vascular anastomoses) [1]. The air that enters the liver graft can cause obstruction to the hepatic microcirculation, obstruction to cardiac output, and paradoxical air embolism producing potential life-threatening systemic effects.

The volume of air as well as rate at which the air is entrained usually determines the morbidity and mortality of air embolisms [4]. This is dependent on a pressure gradient and the caliber of the vessel that entrains the air. The CVP was intentionally kept low-normal during the preanhepatic phase which may have facilitated the air entrainment. Typically, when the air is suctioned by a gravitational gradient, the rate and volume of air entrained are affected by the position of the patient and height of the vein with respect to the right side of the heart. In adults, lethal volume is between 200 and 300 ml, or 3–5 ml/kg. If a large amount of air enters the right side of the heart, an air-lock mechanism may occur, leading to RV outflow obstruction.

There have been numerous reports of large air embolism that had no clinical repercussions or were resuscitated successfully. However, air embolisms usually trigger different cardiovascular, pulmonary, and neurological problems. Electrocardiogram (ECG) abnormalities such as tachyarrhythmias and ST and T wave changes can occur. Our patient initially had a tachycardic response possibly due to right heart strain from the air embolism and hypotension. Additionally, the blood pressure dropped possibly due to the reduction of cardiac output. The PA pressure increased due to both a reduction in cardiac output and increased filling pressures [3]. This sudden decrease in cardiac output causes a ventilation to perfusion mismatch, precipitating a drop in the patient's end-tidal carbon dioxide, arterial oxygen tension and saturation. Transesophageal echocardiography is considered to be the most sensitive monitor for air embolism. TEE may be useful in identifying the size and location of the air embolism as well as intracardiac shunts that allow passage of the air into the arterial circulation [5]. This modality was not used during this case.

Management of venous air embolism focuses on prevention of further air entrainment, reduction of the air entrained and hemodynamic support [4]. We promptly notified the surgeon, and the surgical field was flooded with saline to prevent further entrainment and the defect in the vena cava was also immediately repaired. During that time, we supported the patient's blood pressure with vasopressors. In an effort to reduce the volume of air entrained we aspirated air from the Swan-Ganz catheter. However, both multilumen catheters and Swan-Ganz catheters have a success rate of aspirating air between 6 and 16 percent [4]. These strategies resulted in prompt improvement in the patient's hemodynamics. We suspected that the renal failure that developed was possibly caused by a combination of hypovolemia and venous congestion from the venous air embolism.

In summary, this case report highlights the occurrence of a venous air embolism during an OLT causing hemodynamic instability. Our treatment focused on identifying and repairing the source of entrainment, reduction of volume of air in the right heart through aspiration, and hemodynamic support.
